# Percutaneous uniportal full-endoscopic surgery for treating symptomatic lumbar facet joint cysts under local anesthesia combined with monitored anesthesia care: a preliminary report of eight cases with at least 1 year follow-up

**DOI:** 10.3389/fneur.2023.1278562

**Published:** 2023-12-07

**Authors:** Haining Tan, Lingjia Yu, Xiang Li, Yong Yang, Bin Zhu

**Affiliations:** Department of Orthopedics, Beijing Friendship Hospital, Capital Medical University, Beijing, China

**Keywords:** uniportal endoscopy, full endoscopy, lumbar facet joint cyst, interlaminar approach, transforaminal approach, local anesthesia

## Abstract

**Background:**

Lumbar facet joint cysts (FJCs) are a relatively rare clinical pathology that can result in radiculopathy or neurogenic claudication. Various treatments such as percutaneous aspiration and surgery have been reported to have good clinical outcomes. However, few clinical studies have aimed to treat symptomatic lumbar FJCs by using uniportal full-endoscopic (UFE) surgery. This study aimed to investigate the preliminary clinical outcomes of UFE surgery for the treatment of lumbar FJCs under local anesthesia combined with monitored anesthesia care (MAC).

**Methods:**

Eight patients (five males and three females) with symptomatic lumbar FJCs who underwent UFE surgery under local and MAC anesthesia were enrolled in this study between January 2018 and April 2022. The clinical characteristics, radiological features, operative information, visual analog scale (VAS) score, Oswestry disability index (ODI), and overall outcome rating based on the modified MacNab criteria were retrospectively analyzed.

**Results:**

Of the eight patients, four underwent a transforaminal approach and four underwent an interlaminar approach. Postoperatively, the mean VAS score for leg pain decreased from 6.1 before surgery to 0.6 after surgery, and the ODI decreased from 74.5% to 14.7%. All patients were followed up for more than 1 year, and the good-to-excellent rate based on the modified MacNab criteria remained 100% at the last follow-up. No complications occurred during the follow-up period.

**Conclusion:**

Lumbar FJCs can cause severe radiating leg pain and/or neurogenic claudication due to the dural sac compression and nerve roots. As an alternative treatment, UFE decompression under local and MAC anesthesia may provide effective clinical outcomes for symptomatic lumbar FJCs.

## 1 Introduction

Facet joint cysts (FJCs) are estimated to affect 0.65%–6.4% of the population and are commonly found in the lumbar spine but rarely in the cervical or thoracic spine (1–5). The pathogenesis of FJCs remains unknown; however, FJCs are associated with spinal instability or degenerative spondylolisthesis (6, 7). Lumbar FJCs can be symptomatic because of compression of the spinal cord or nerve roots, resulting in radiculopathy, back pain, cauda equina syndrome, or neurological claudication, especially in cases of hematoma of the cysts, which occurs in approximately 3% of synovial cysts (8–11).

Image-guided intraarticular aspiration, image-guided cyst rupture, image-guided epidural steroid injection, laminotomy, and decompression by microsurgical or traditional open techniques have been reported to be effective in treating symptomatic lumbar FJCs (12–16). Recently, uniportal full endoscopy (UFE), a minimally invasive technique, has been widely applied to treat lumbar disc herniation, lumbar spinal stenosis, and cervical spondylopathy (17, 18). Moreover, the UFE technique has also been used for the treatment of lumbar FJCs, showing satisfactory clinical and radiological outcomes (19, 20). However, only a few studies have reported on the UFE technique for treating lumbar FJCs under local anesthesia (21, 22). Therefore, this study aimed to investigate the preliminary clinical outcomes of the UFE technique for the treatment of symptomatic lumbar FJCs under local anesthesia combined with monitored anesthesia care (MAC).

## 2 Methods

### 2.1 Subjects

This retrospective study was approved by the Ethics Committee of the Beijing Friendship Hospital, Capital Medical University. Informed consent was obtained from all the participants. The medical records of patients with symptomatic lumbar FJCs who were hospitalized at our spine center and underwent surgical resection using the UFE technique by one senior spine surgeon between January 2018 and April 2022 were retrospectively reviewed. The inclusion criteria were as follows: (1) neurogenic claudication or radiating leg pain with associated neurological signs; (2) compression by FJCs confirmed on the lumbar MRI; and (3) failure of conservative treatment for at least 3 months. Three patients were excluded due to ≥II degree lumbar spondylolisthesis, a prior history of surgery at the surgical level, and incomplete medical records.

### 2.2 Surgical technique

All procedures were performed under local anesthesia, MAC, and sufentanil administration. All procedures were performed with endoscopic techniques through an interlaminar or transforaminal approach. The surgical approach for UFE was decided based on the anatomical locations of the FJCs. The interlaminar approach was performed in the other four patients because the FJCs were located around the caudal half of the medial side of the facet, and the transforaminal approach was used in four patients because the FJCs were located around the rostral half of the medial side of the facet.

Patients who underwent UFE surgery using the interlaminar approach were placed in the prone position on a radiolucent table. An 18-gauge spinal needle was inserted into the posterior ligamentum flavum, and the needle was confirmed to be located at the midpoint of the interlaminar space of the fluoroscope. The guidewire, obturator, working cannula, endoscopic trephine, and endoscopic system were then inserted sequentially. The ipsilateral upper lamina, inferior articular process, and superior articular process were partially removed using a trephine or Kerrison punch to adequately expose the facet cyst. After opening the ligamentum flavum, the facet cysts were detected and removed using Kerrison punches or disc forceps. Surgeries were concluded when the complete resolution of dura and nerve root compression had been obtained with non-evidence of the residual cyst wall.

**Figure 1:**
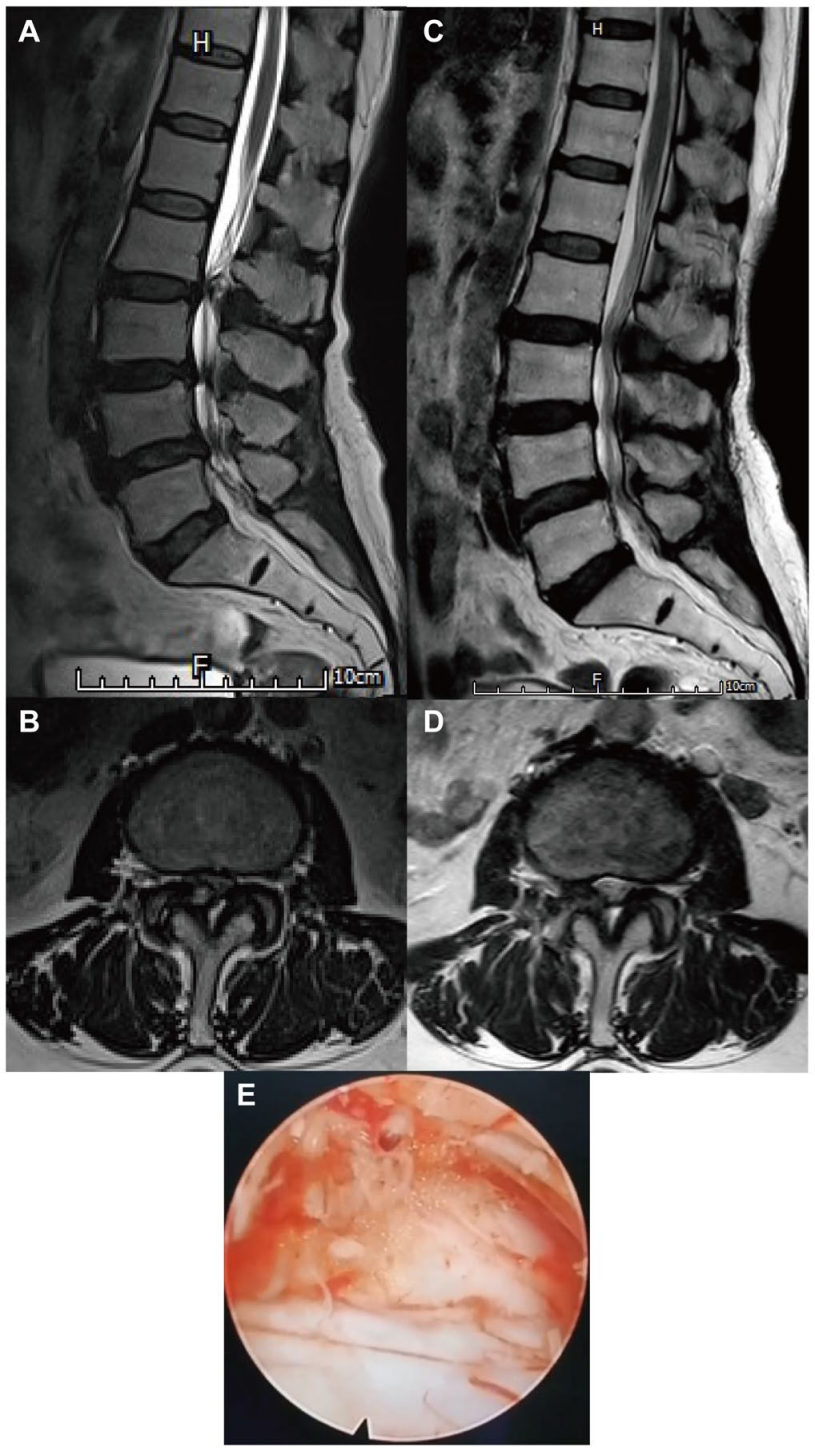
Radiological images of a patient with lumbar facet joint cyst (FJC) who underwent uniportal full-endoscopic (UFE) surgery through a transforaminal approach. **(A,B)** Preoperative magnetic resonance imaging (MRI) of the lumbar spine revealed one cyst located at the right facet joint at the L2-3 level. **(C,D)** A postoperative MRI of the lumbar spine showed that the FJC was completely removed. **(E)** Endoscopic view of the FJC during the UFE surgery.

Patients who underwent UFE using the transforaminal approach were laterally positioned on a radiolucent table. An 18-gauge spinal needle was inserted into the lateral aspect of the superior articular facet, as confirmed by fluoroscopy. The guidewire, obturator, working cannula, endoscopic trephine, and endoscopic system were inserted sequentially. The superior articular process was partially resected using a trephine. The facet cyst was resected using disc forceps. Surgeries were concluded when the complete resolution of dura and nerve root compression had been obtained with non-evidence of the residual cyst wall (Figure 1).

### 2.3 Demographic data collection and outcome assessment

Demographic data including sex, age, surgical level, operation time, estimated blood loss, duration of postoperative hospitalization, and complications were systematically collected. Visual analog scale (VAS) scores for leg pain were evaluated preoperatively, immediately postoperatively, and at the last follow-up (at least 1 year). Oswestry disability index (ODI) scores were assessed preoperatively and at the last follow-up. The modified MacNab criteria were recorded at the last follow-up (23). Computed tomography (CT) of the lumbar spine was performed preoperatively and 1 day postoperatively. Magnetic resonance imaging (MRI) of the lumbar spine was performed before surgery and at 3 months follow-up.

### 2.4 Statistical analysis

Categorical variables were grouped and presented as numerical values, whereas continuous data were presented as mean values. The Wilcoxon signed-rank test was used to compare VAS and ODI scores at the preoperative, postoperative, and last follow-up. Statistical significance was set at a *p*-value of <0.05. All data analyses were performed using SPSS v25.0 software (IBM Corp., Armonk, NY, United States).

## 3 Results

### 3.1 Characteristics of patients and facet cysts

Eight patients with lumbar FJCs were enrolled in this study, five male and three female patients. The mean age was 53.9 years (range 28–72 years). All eight patients had radiating leg pain as the chief complaint, of whom two had neurogenic claudication. Based on preoperative MRI of the lumbar spine, most facet cysts (7/8, 72.5%) were located at the L4-5 level, and only one cyst was at the L2-3 level. The mean duration of operation was 72.0 min (range 60–90 min), the mean estimated blood loss was 31.2 mL (range 20–50 mL), and the length of postoperative stay was 1.5 days (range 1–3 days) (Table 1).

**Table 1:** Patients’ information.

Patient	Sex	Age	Symptom	Facet cyst location	Approach	Operation duration (min)	Estimated blood loss (mL)	Length of postoperative stay (days)
1	Male	63	Radiculopathy	Left L4-5	Transforaminal	60	25	1
2	Male	45	Claudication + Radiculopathy	Left L4-5	Interlaminar	68	20	1
3	Female	62	Radiculopathy	Left L4-5	Transforaminal	72	30	2
4	Male	39	Radiculopathy	Left L4-5	Transforaminal	72	25	2
5	Male	28	Claudication + Radiculopathy	Left L4-5	Interlaminar	65	30	1
6	Female	72	Radiculopathy	Right L2-3	Interlaminar	90	20	2
7	Female	57	Radiculopathy	Right L4-5	Transforaminal	65	50	2
8	Male	65	Radiculopathy	Left L4-5	Interlaminar	84	50	1

### 3.2 Surgical outcomes

All patients were followed up for at least 12 months (range 12–23 months). The mean VAS score for leg pain significantly improved from 6.1 (range 5–7) to 0.6 (range 0–2) immediately after surgery (*p* = 0.010) and further decreased to 0.5 (range 0–2) at the last follow-up (*p* = 0.010). The mean ODI score at the last follow-up significantly improved from 74.5% (range 66%–80%) to 14.7% (range 8%–25%) compared to the preoperative score (*p* = 0.012). Based on the modified MacNab criteria at the last follow-up, excellent results were obtained in six (75%) patients and good results in two (25%) patients. No perioperative complications were observed, including dural tears, neurologic deterioration, or hematoma compression, and no spinal instability or reoperation was performed during follow-up. No residual cyst was detected by postoperative CT or MRI (Table 2).

**Table 2:** Surgical outcomes.

Patient	VAS for leg pain			ODI		MacNab	Follow-up (month)	Complication
	Preoperative	Postoperative immediately	Last follow-up	Preoperative	Last follow-up			
1	7	2	2	75	25	Good	23	N/A
2	6	1	0	68	12	Excellent	20	N/A
3	5	0	0	78	8	Excellent	14	N/A
4	7	2	2	80	30	Good	16	N/A
5	7	0	0	66	12	Excellent	18	N/A
6	6	0	0	71	16	Excellent	12	N/A
7	5	0	0	78	8	Excellent	12	N/A
8	6	0	0	80	15	Excellent	12	N/A

VAS, visual analog scale; ODI, Oswestry disability index.

## 4 Discussion

Percutaneous aspiration, rupture, corticosteroid injection, and surgery were effective in patients with symptomatic lumbar FJCs who failed to respond to conservative therapy. Kim et al. (14) evaluated the clinical outcomes of a three-stage minimally invasive percutaneous technique for lumbar intraspinal synovial cysts and found that endoscopic superior facetectomy resulted in no recurrence within the 3 years follow-up. One meta-analysis revealed no significant differences in outcome or complication rates between microscopic and endoscopic resection for lumbar FJCs (13). Another meta-analysis showed that full endoscopy could achieve 90% satisfactory outcomes with low rates of adverse events (<2%) better than open and minimally invasive surgeries (24). One prospective multicenter study by Tacconi et al. (20) reported the clinical outcomes of full-endoscopic surgery for lumbar FJCs. At a median follow-up of 15 months, 89% of patients were pain-free or improved, showing outcomes comparable to those of open or tubular techniques. Hellinger et al. (25) analyzed 2 years follow-up outcomes of 48 patients who were treated with endoscopic removal of extradural cysts; excellent or good results based on the MacNab criteria were obtained in 37 out of 48 (77.1%) patients. Hagan et al. (22) introduced awake transforaminal endoscopic decompression surgery for the treatment of lumbar FJCs in patients with lumbar radiculopathy, and the VAS score for leg pain improved significantly without complications, readmission, or symptom recurrence during a 2 years follow-up period. In this study, the VAS score for leg pain and ODI score improved after UFE surgery. The overall excellent-good rate of the modified MacNab criteria was 100% without any complications or recurrence. These findings support the satisfactory outcomes achieved using the UFE technique in patients with symptomatic lumbar FJCs. Moreover, the UFE approach was chosen mainly based on the location of the lumbar FJCs relative to the facet joints. When the FJCs were located around the rostral half of the medial side of the facet, the transforaminal approach was preferred; otherwise, the interlaminar approach was considered (21). In addition, the choice of surgical approach should consider the operating habits of the surgeon and the anatomical characteristics of the patient’s lumbar spine.

Whether fusion is needed has also been discussed in patients with lumbar FJCs undergoing decompressive operation (26). The NeuroSpine Surgery Research Group proposed a classification system (grades 1–5) for lumbar FJCs based on the percentage of the vertebral canal occupied by the cyst on MRI and the degree of spondylolisthesis in the involved segment. Patients with grade 4 and 5 FJCs show a greater risk of recurrence following decompression alone, and stabilization of the involved segments should be considered for initial decompressive surgery in these patients (27, 28). Thompson et al. (29) reported that the revision rate was as high as 20.4% in patients who received limited decompression for lumbar FJCs and found that a facet angle >45° at L4-5 was associated with the risk of failure of primary decompression. Page et al. (30) reported a predictive model for lumbar synovial cyst recurrence following decompression without fusion and found some predictive factors of recurrence, including a facet inclination angle of >45°, canal stenosis of >50%, T2 joint space hyperintensity, and the presence of grade I spondylolisthesis. One systematic review and meta-analysis showed that decompression combined with fusion was associated with better results in terms of lower postoperative back pain and cyst recurrence compared with decompression alone; however, there were no differences in the reoperation and complication rates (31). None of the patients in this study experienced recurrence or reoperation during the follow-up period. This may be associated with the advantage of the UFE technique of minimal trauma to the paraspinal muscles and the posterior ligamentous complex of the lumbar spine (32).

The current study had some limitations. First, it was a retrospective study with a relatively small sample size. Further prospective studies with larger sample sizes are warranted to verify the efficacy of UFE. In addition, the minimum 1 year follow-up was relatively short to assess recurrence and reoperation. A continuous long-term follow-up of these patients is needed.

Lumbar FJCs can cause severe radiating leg pain and/or neurogenic claudication due to the compression of the dural sac and nerve root. The decompression accomplished by the UFE technique under local and MAC anesthesia may provide effective clinical outcomes for symptomatic lumbar FJCs and could be an alternative treatment for symptomatic lumbar FJCs.

## Data availability statement

The original contributions presented in the study are included in the article/supplementary material, further inquiries can be directed to the corresponding authors.

## Ethics statement

The studies involving humans were approved by Ethics Committee of Beijing Friendship Hospital, Capital Medical University. The studies were conducted in accordance with the local legislation and institutional requirements. Written informed consent for participation in this study was provided by the participants’ legal guardians/next of kin. Written informed consent was obtained from the individual(s) for the publication of any potentially identifiable images or data included in this article.

## Author contributions

HT: Conceptualization, Funding acquisition, Writing – original draft. LY: Investigation, Writing – original draft. XL: Supervision, Writing – original draft. YY: Supervision, Writing – original draft. BZ: Conceptualization, Funding acquisition, Writing – review & editing.
